# Supplementary figures and images for: Stress-induced dysfunction of neurovascular astrocytes contributes to sex-specific behavioral deficits
Source: bioRxiv. 2024 May 14:2024.05.14.594147. Preprint. [Version 1] doi: 10.1101/2024.05.14.594147 (PMC11118421; doi:10.1101/2024.05.14.594147)

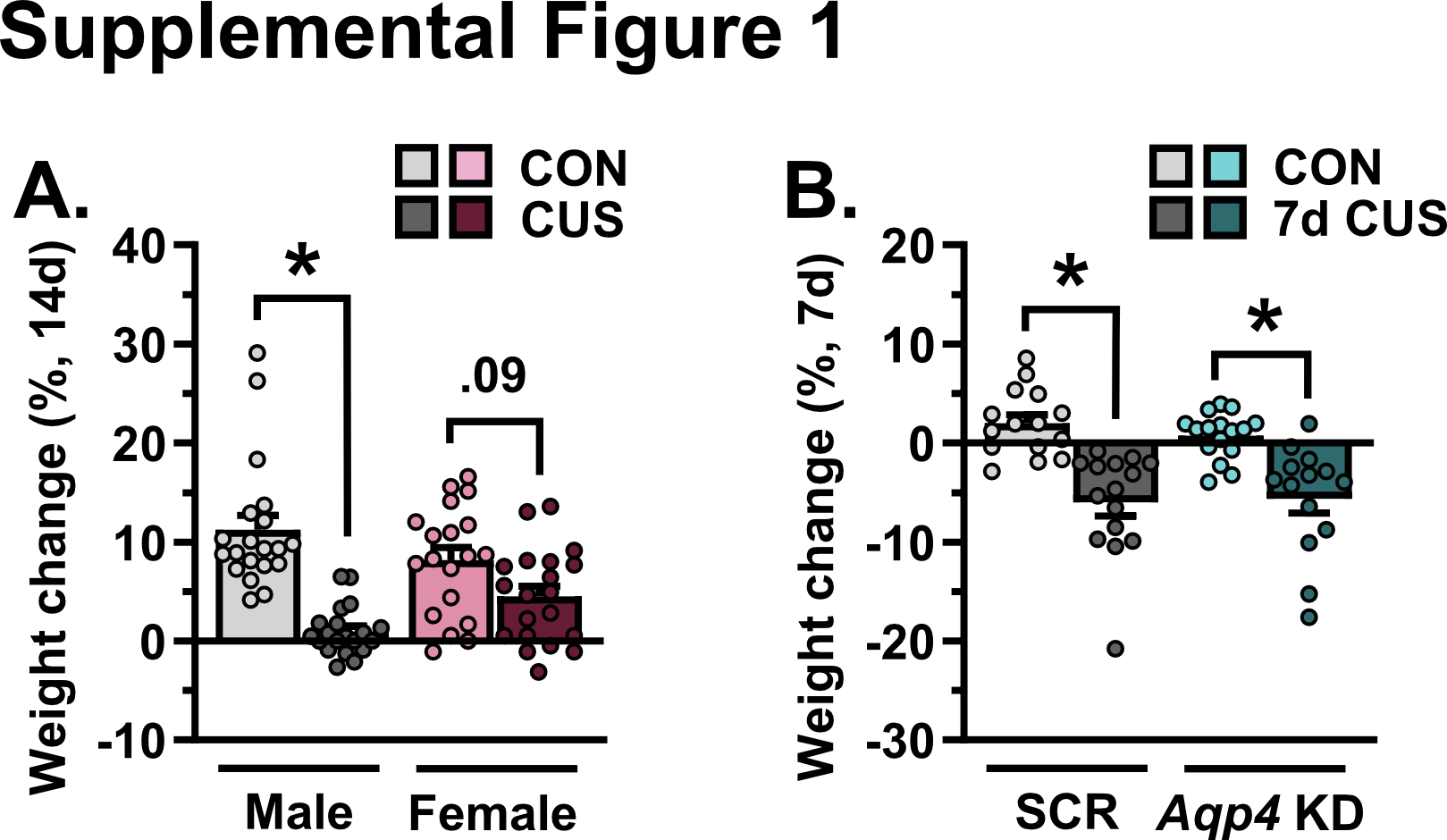

Supplement: Supplement 1 — Supplementary Figure 1. Chronic stress reduces animal weight gain. A) Weight change in mice subjected to CUS or left unstressed (14-days). Stress differentially reduced weight gain in male and female mice (F(1,75)=8.148 p=0.005). Planned comparisons indicate a significant reduction in weight gain in stressed males (p<0.0001) and a trend toward this in stressed females (p=0.089). B) Weight change in mice subjected to sub-CUS or left unstressed (7-days). Exposure to sub-CUS reduced weight gain in mice, regardless of Aqp4 knockdown in the PFC (F(1,26)=41.98 p<0.001). Bars represent mean ± S.E.M. * p<0.05 comparison indicated (Sidak’s test). [file media-1.jpg]

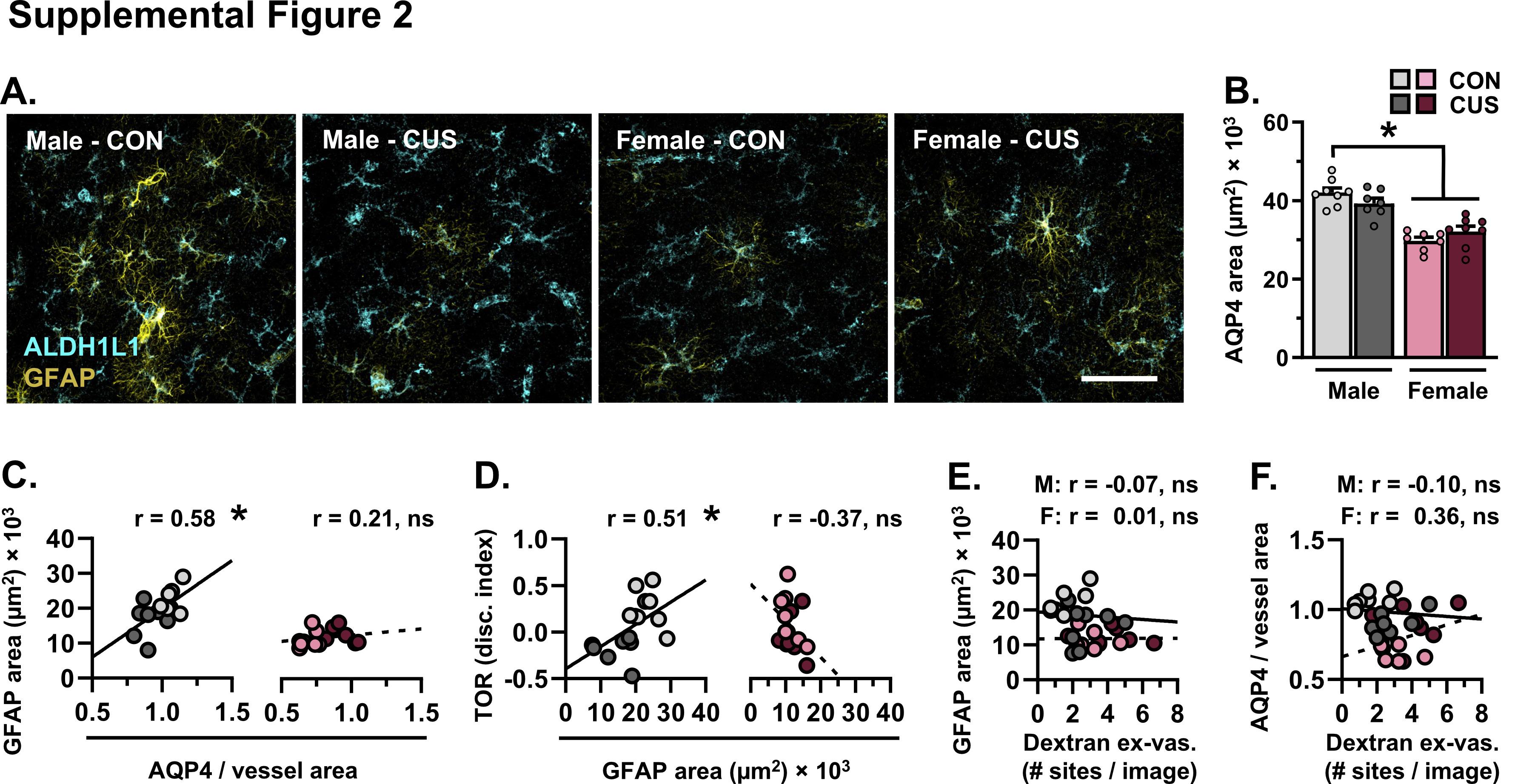

Supplement: Supplement 2 — Supplementary Figure 2. Loss of astrocytic structures is associated with diminished aquaporin-4 blood vessel coverage in the prefrontal cortex and working memory impairment in male mice. A) Representative images of ALDH1L (Cyan) and GFAP (yellow) immunohistology in the PFC (20×, scale bar = 50 μm. B) Total area of AQP4+ material in the PFC. C) Linear association between GFAP+ area and AQP4 blood vessel coverage in the PFC. D) Linear association between GFAP+ area in the PFC and discrimination in the TOR. E) Linear association between GFAP+ area and the number of dextran extravasation sites in the PFC. F) Linear association between AQP4 blood vessel coverage and the number of dextran extravasation sites in the PFC. Bars represent mean ± S.E.M. * p<0.05 comparison indicated (Sidak’s test or Pearson’s correlation). [file media-2.jpg]

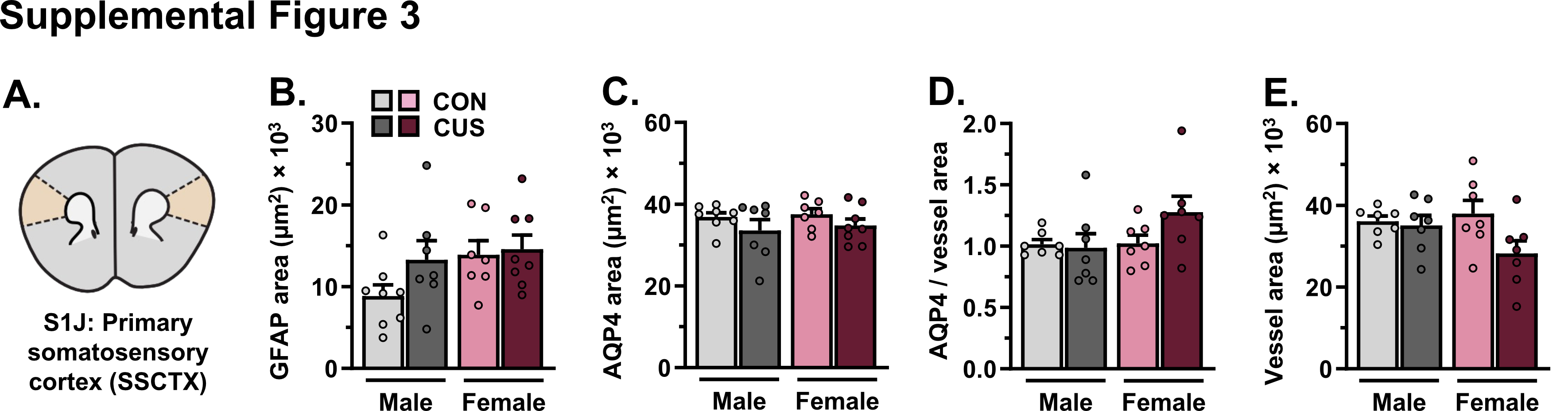

Supplement: Supplement 3 — Supplementary Figure 3. Analysis of astrocyte coverage and blood vessel area in the somatosensory cortex. A) Schematic of the S1J subregion of the SSCTX. Analysis of this region occurred in the same animals as presented in Figure 3. B) Area of GFAP+ material in the SSCTX. C) Total area of AQP4+ material in the SSCTX. D) Area of astrocyte AQP4+ material relative to vessel area. E) Area of tomato lectin+ vessels in the SSCTX. Bars represent mean ± S.E.M. [file media-3.jpg]
